# Adipose tissue plays a major role in retinoic acid-mediated metabolic homoeostasis

**DOI:** 10.1080/21623945.2021.2015864

**Published:** 2021-12-26

**Authors:** Shenglong Zhu, Jingwei Zhang, Doudou Zhu, Xuan Jiang, Lengyun Wei, Wei Wang, Yong Q. Chen

**Affiliations:** a Wuxi School of Medicine, Jiangnan University, Wuxi, China; b Wuxi Translational Medicine Research Center and Jiangsu Translational Medicine Research Institute Wuxi Branch, China; c School of Food Science and Technology, Jiangnan University, Wuxi, China

**Keywords:** Non-alcoholic fatty liver disease, Retinoic acid, Adipose tissues

## Abstract

Retinoic acid (RA), a bioactive metabolite of vitamin A, has shown therapeutic effects in liver disease, and its effect in improving non-alcoholic fatty liver disease (NAFLD) is associated with the inhibition of adipogenesis in the white adipose tissue (WAT) and fatty acid oxidation induction in the liver. However, the major target organ of RA is unknown. We performed chronic administration of RA in high-fat diet (HFD)-induced NAFLD mice. Further, hepatic and adipose cells were used to study the direct effect of RA on lipid metabolism. In addition, qRT-PCR was performed to examine differential gene expression in mouse adipose tissue. RA administration ameliorated NAFLD in HFD-induced obese mice and increased mouse energy expenditure. Although RA had therapeutic effects on liver histology and lipid accumulation, it did not directly affect lipid metabolism in HepG2 cells. In contrast, RA reduced the weight of several adipose tissues and improved lipid accumulation in OP9 cells. In addition, RA upregulated genes responsible for fatty acid oxidation and thermogenesis in three different WATs. Our work suggests that the liver may not be the main target organ of RA during NAFLD treatment. WAT browning induced by RA may be the primary contributor towards the amelioration of NAFLD in HFD-induced obese mice.

## Introduction

Non-alcoholic fatty liver disease (NAFLD) represents a spectrum of hepatic pathologies, ranging from simple steatosis to non-alcoholic steatohepatitis (NASH) and cirrhosis [1]. NAFLD is frequently associated with obesity and diabetes mellitus, and NAFLD development is strongly linked to metabolic syndrome [2]. However, no approved therapy for NAFLD currently exists.

Retinoic acid (RA), a major metabolite of vitamin A, has many remarkable effects on lipid and energy metabolism [3,4]. RA treatment significantly reduces body weight and adiposity independent of changes in food intake and improves insulin sensitivity and glucose tolerance in high-fat diet (HFD) induced NAFLD mice [5].

The pathogenesis of NAFLD is complex and relies on the crosstalk between liver and adipose tissues [5]. Studies have demonstrated that the therapeutic effect of RA in improving NAFLD is associated with the inhibition of adipogenesis in the white adipose tissue (WAT) and the induction of fatty acid oxidation in the liver [4,6,7]. However, the organ that is the major target of RA is still unclear.

To address this issue, we performed chronic administration of RA in HFD-induced NAFLD and explored the relationship between lipid metabolism in the liver and that in eight different parts of the adipose tissue. Furthermore, we used hepatic and adipose cells to study the direct effect of RA on lipid metabolism. In this study, we found that adipose cells played a major role in RA-mediated metabolic homoeostasis and RA protected against HFD-induced NAFLD mainly by WAT browning.

## Results

### RA administration improves lipid and glucose homoeostasis

**Figure 1.**
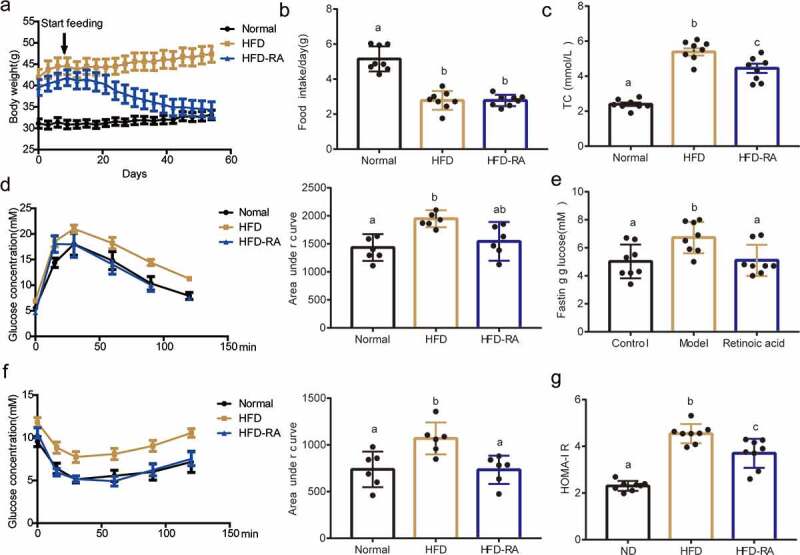
**Retinoic acid protects against high-fat diet-induced lipid and glucose changes to maintain homoeostasis**. Effects of RA treatment in WT mice fed with HFD. Reduction in body weight (a) and Food intake (b) plasma TC (c), Glucose tolerance test (d), and insulin tolerance test (f) were performed in WT mice fed with HFD and treated with RA (HFD-RA). (e) Fasting glucose. (g) HOMA-IR. Data are shown as mean ± SEM. One-way ANOVA was performed to compare multiple groups, and differences (P < 0.05) have been labelled with different letters; same letters mean no significance (P > 0.05)

Previously published data in rodents demonstrates that RA prevents and/or reverses diet-induced obesity and insulin resistance [8]. To investigate the mechanisms of these protective effects of RA, C57BL/6 J mice were fed with or without RA in high-fat diets (HFDs). Results demonstrated that RA treatment for 8 weeks decreased the body weight of HFD-fed mice (Figure 1a) and no significant change in food intake was observed (Figure 1b). Compared with mice in the HFD group, those in HFD-RA group showed substantially decreased TC levels in the plasma (Figure C). Moreover, in glucose tolerance test (GTT) and insulin tolerance test (ITT), mice in the HFD-RA group showed improved glucose tolerance and insulin sensitivity (Figure 1d,F). Also, fasting glucose and HOMA-IR were significantly reduced in the HFD-RA group (Figure 1e,G). These results showed that RA treatment improved the metabolic function.

### RA administration increases energy expenditure

**Figure 2.**
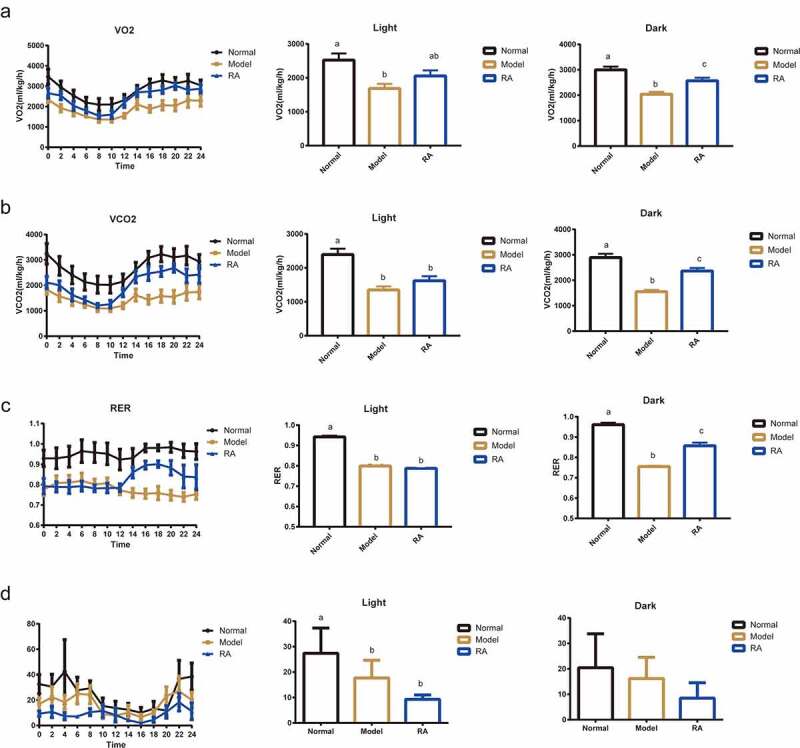
**Retinoic acid enhances energy expenditure**. (a-d) the curve of oxygen consumption rate (VO_2_), carbon dioxide production rate (VCO2), respiratory exchange ratio (RER), and mice activity during a 24-h period. Average VO_2_, VCO2, and RER in both light and dark cycles. The energy expenditure was monitored using CLAMS for 72 h. Data are shown as mean ± SEM. One-way ANOVA was performed to compare multiple groups, and differences (P < 0.05) have been labelled with different letters; same letters indicate no significance (P > 0.05)

Increasing energy expenditure is an important mechanism to prevent obesity [9]. We tested the effect of RA on animal energy expenditure using a laboratory animal monitoring system (CLAMS). The metabolic analysis showed increased O_2_ consumption, CO_2_ production, and respiratory exchange ratio (RER) in the HFD-RA group compared with the HFD group during dark period (Figure 2a-C). Importantly, the protective effect of RA was associated with a significant increase in energy expenditure as evaluated by the oxygen consumption test. Further, the values of respiratory metabolism parameters were higher in the HFD-RA group than in the HFD group mainly in the dark period. These data demonstrated that the increased energy expenditure induced by RA could be the primary contributor for the prevention of obesity in NAFLD mice.

### The liver may not be the target of RA in NAFLD treatment

**Figure 3.**
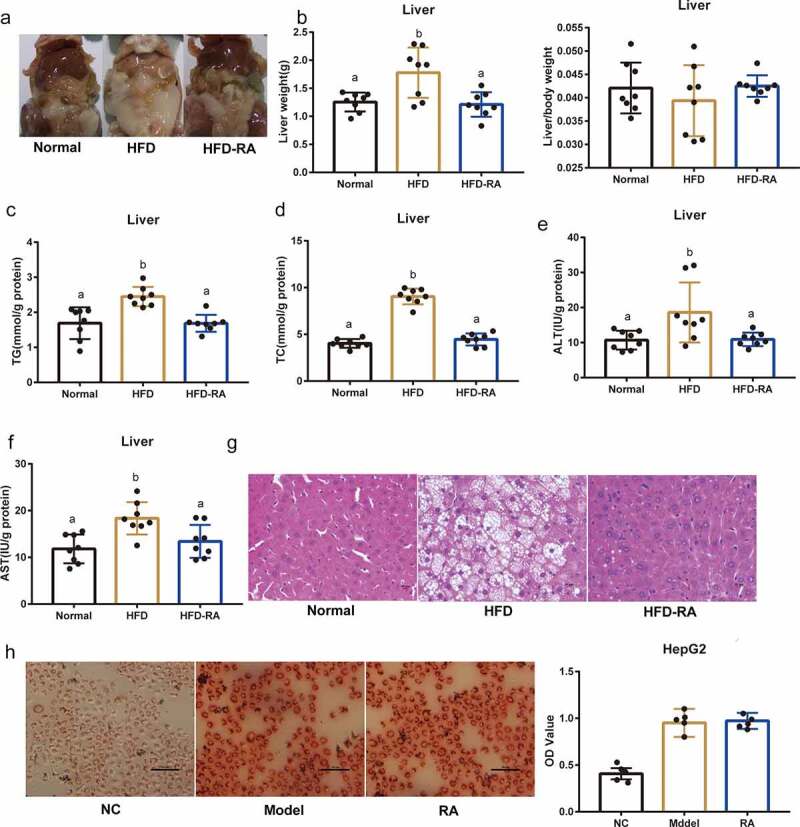
**Amelioration of non-alcoholic fatty liver disease by retinoic acid does not directly depend on the liver**. (a) Representative photograph of the liver. (b) liver weight and liver/body weight. (c-f) Hepatic TG, TC, ALT, and AST levels. (g) H&E staining of the liver. (h) The effects of RA on OA- and PA-induced lipid accumulation in HepG2 cells. Intracellular lipid accumulation was measured by Oil Red O staining using an inverted microscope. The OD at 510 nm was measured. Data are shown as mean ± SEM. One-way ANOVA was performed to compare multiple groups, and differences (P < 0.05) have been labelled with different letters; same letters indicate no significance (P > 0.05)

Treatment with RA significantly reduced the size of the liver and the degree of fat deposition in the liver (Figure 3a). However, there was no significant difference in the liver to body weight ratio between HFD-RA and HFD groups (Figure 3b). RA treatment decreased hepatic TG and TC levels (Figure 3c,D). In addition, RA reduced intracellular hepatic ALT and AST levels (Figure 3e,F). Moreover, fatty changes in the liver, as evaluated by H&E staining, were reduced after treatment with RA in NAFLD mice (Figure 3g). HepG2 cells were used to investigate whether the therapeutic effects of RA in improving metabolic disorders were directly through targeting the liver. However, oil red O staining showed that RA had no effect on lipid metabolism in HepG2 cells (Figure 3h). These data suggest that the therapeutic effects of RA in improving metabolic disorders may not directly be through targeting the liver.

### RA exerts therapeutic effects by targeting the adipose tissue

**Figure 4.**
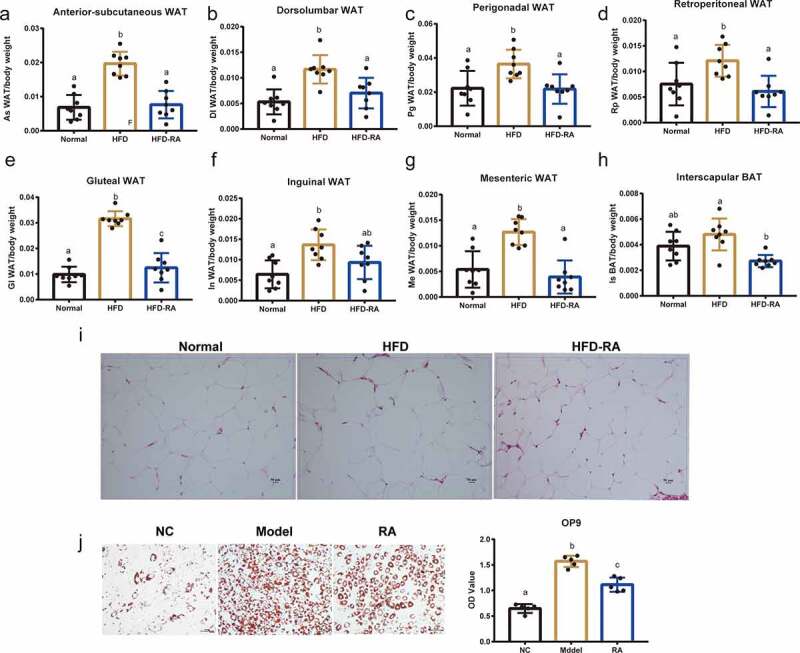
**White adipose tissue plays an important role in non-alcoholic fatty liver disease mice treated with retinoic acid**. (a) Anterior-subcutaneous white adipose tissue (WAT)/body weight. (b) Dorsolumbar WAT/body weight. (c) Perigonadal WAT/body weight. (d) Retroperitoneal WAT/body weight. (e) Gluteal WAT/body weight. (f) Inguinal WAT/body weight. (g) Mesenteric WAT/body weight. (h)Interscapular BAT/body weight. (i) H&E staining of WAT. (j) The effects of RA on OA- and PA-induced lipid accumulation in OP9 cells. Intracellular lipid accumulation was measured by Oil Red O staining using an inverted microscope. The OD at 510 nm was measured. Data are shown as mean ± SEM. One-way ANOVA was performed to compare multiple groups, and differences (P < 0.05) have been labelled with different letters; same letters indicate no significance (P > 0.05)

WAT functions primarily as a key regulatory centre of energy metabolism and a site for fuel storage [10]. Considering the increased energy expenditure induced by RA (Figure 2), we then evaluated the effects of RA on WAT. In general, WAT is categorized as subcutaneous WAT, dorsolumbar WAT, perigonadal WAT, retroperitoneal WAT, gluteal WAT, inguinal WAT, and mesenteric WAT. Unlike our results observed in the liver, treatment with RA significantly reduced the weight of different WATs and interscapular brown adipose tissue (BAT) in NAFLD mice (Figure 4a-H). Furthermore, as shown in H&E staining, RA treatment reversed the apparent fat cell changes caused by HFD (Figure 4i). In addition, on using the OP9 differentiation model, we found that RA significantly affected adipose differentiation (Figure 4j). These data showed that RA treatment had a direct effect on WAT, and changes in WAT by RA could be the primary contributor towards NAFLD amelioration.

### RA upregulated fatty acid oxidation genes and thermogenesis genes in WAT in NAFLD mice

**Figure 5.**
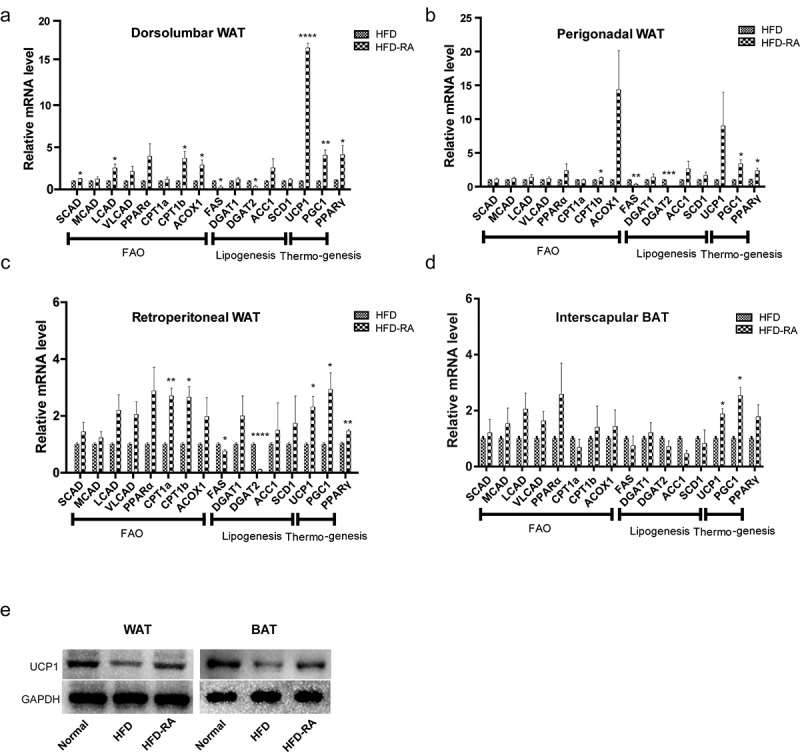
**Retinoic acid enhances the transcription of thermogenic programming genes**. quantitative reverse transcription polymerase chain reaction (qRT-PCR) was performed to show the expression of genes associated with fatty acid oxidation and thermogenesis in three WATs and BATs (a-d). Protein levels of UCP1 were detected by Western blot analysis (e). Data are presented as mean ± SEM. Unpaired two-tailed Student’s t-test was conducted to compare between two groups and significance has been marked with * (P < 0.05), ** (P < 0.01), *** (P < 0.001), and **** (P < 0.0001)

In an attempt to unveil the mechanisms by which RA affects adipose, we assessed the effect of RA on the expression of genes that control energy expenditure and thermogenic programing in different WATs and interscapular BAT. Compared with mice in the HFD group, those in the HFD-RA group showed increased transcription levels of carnitine palmitoyltransferase 1b (*CPT1B*) and acyl-CoA oxidase 1 (*ACOX1*), which are essential genes for controlling fatty acid oxidation. Moreover, the expression levels of thermogenesis genes including uncoupling protein 1 (*UCP1*) and peroxisome proliferator activated receptor gamma (*PPARγ*); markers of adipose tissue browning; and Peroxisome proliferator-activated receptor gamma coactivator 1 *(PGC1*), a key thermogenic transcriptional factor, were upregulated (Figure 5a-D). Further study showed that the protein levels of UCP1 were significantly increased in both white adipose and brown adipose tissues (Figure 5e). These data suggest that RA promotes thermogenesis by enhancing the transcription of thermogenic programming genes and inducing WAT browning.

## Discussion

The adipose tissue is a mediator of metabolism and has been implicated in the development of NAFLD [11]. RA signalling acts as a central regulator in adipose tissue remodelling [12] and is well known to induce adipocyte differentiation. In previous research, browning of inguinal subcutaneous and perigonadal visceral WATs was found to be associated with reduced obesity [13]. Here, we detected the effect of RA on eight different adipose tissues including visceral and subcutaneous adipose tissues [14]. As expected, except for inguinal WAT, the weight of other WATs significantly decreased after RA treatment. Besides, lipid accumulation in OP9 cells was decreased after RA treatment. This finding is also in agreement with other findings that RA upregulates the mRNA levels of *UCP1*, transcription factors (*PGC-1, PPARγ*), and enzymes (*CPT1*) involved in fatty acid oxidation in different WAT depots, and is consistent with the finding of an increased capacity for oxidative metabolism/thermogenesis in these depots [6]. Taken together, these results indicate that RA may protect against HFD-induced NAFLD by WAT browning.

The receptors of RA, i.e., RA receptors (RARs) and retinoid X receptors (RXRs), are two transcription factors mediating RA’s ability to regulate gene expressions [15]. A previous study showed that reduction in RXR signalling appears to attenuate HFD-induced obesity and diabetes mellitus type 2 [16], and overexpression of RXR elevates glucose disposal. Additionally, several studies suggest that RA activity is mediated primarily by members of the RAR subfamily, namely RARα, RARβ, and RARγ, which belong to the nuclear receptor superfamily of transcription factors [17]. RARα and RARγ are highly expressed in WAT depots [18], whereas RARβ is relatively more abundant in the liver [19]. This characteristic expression pattern may indicate specific functions for RAR isoforms in liver and WAT biology. Lee et al. have shown that altered RAR signalling in adipocytes resulted in a significant decrease in all-*trans*-retinoic acid levels in visceral adipose tissues as well as in the liver tissue [20]. In this study, it is evident that RA protected mice from NAFLD. It did not affect lipid metabolism in HepG2 cells, but did affect the metabolism in OP9 cells, which may be because of the differences in RA receptor’s expressions between adipose and liver tissues.

In conclusion, our work suggests that the liver may not the main target of RA during NAFLD treatment. WAT browning induced by RA may be the primary contributor towards the amelioration of NAFLD in the diet-induced obese mice. The receptors that played a key role in RA-induced therapeutics in adipose tissue need further study. Meanwhile, there is still a lot to learn about RA in WATs in healthy and pathological conditions, which hopefully will reveal novel therapeutic targets for NAFLD treatment.

## Materials and methods

### Cell culture and differentiation

OP9 mouse stromal cell and HepG2 cell were obtained from the American Type Culture Collection (ATCC, USA). HepG2 cells were maintained in Dulbecco’s modified Eagle’s medium (DMEM; Life Technologies, Grand Island, NY, USA) supplemented with 10% foetal bovine serum (FBS; Life Technologies). Palmitic acid (PA) and oleic acid (OA) were purchased from Sigma-Aldrich (St. Louis, MO, USA). HepG2 cells were incubated with 300 μM oleic acid (OA) plus 300 μM palmitic acid (PA) for 48 h, as described previously. Subsequently, the medium was replaced with a normal fresh medium containing RA at final concentration of 10 μM and incubation was continued for an additional 48 h. OP9 cells were maintained in MEM-α containing L-Glutamine, with 20% FBS (Secure foetal bovine serum; Gibco, Australia), 100 U/mL penicillin, and 100 μg/mL streptomycin [21]. For adipocyte differentiation, 1 μM rosiglitazone was added to the culture medium for differentiation at 37°C in 5% CO_2_. RA was then added to the medium at 10 μM for 5 days. Three wells of OP9 cells were used for each experiment. After treatment for 5 days, the cells were washed twice with PBS, fixed with 4% paraformaldehyde at room temperature for 30 min, and then stained with oil red O (Sigma, USA). The absorbance was measured at 510 nm.

### Quantitative real-time PCR

Total RNA was extracted using Trizol (Ambion, USA). cDNA was synthesized using a reverse transcription reagent kit (RT Reagent Kit with gDNA Eraser RR047A, Takara, Japan). Gene expression levels were analysed by quantitative real-time PCR using the BIO-RAD CFX Connect Real-Time System (CA, USA).

### HFD feed and metabolic assays

The animal protocols followed in this study were approved by the Animal Ethics Committee of Jiangnan University, China. All experiments were performed in accordance with Chinese regulations for the administration of affairs concerning experimental animals 2017. Male C57BL/6 mice (6 weeks old) were purchased from the Xi Nuo Sai BioScience, Inc. (Suzhou, China) and randomly classified into two groups: normal diet (ND; chow diet, 10% of calories derived from fat) and high-fat diet (HFD, 60% of calories derived from fat, Research Diets, Beijing, China; D12451). After 12 weeks of modelling, the HFD group was further divided into two groups, fed with or without RA (50 mg RA per 1 kg diet). Eight mice were included in each group.

The respiratory exchange ratio (RER, the volume ratio of CO_2_ exhaled versus O_2_ consumed), VO_2_ (mL/kg/h), and VCO_2_ (mL/kg/h) were measured using metabolic chambers (Columbus Instruments, Columbus, OH). GTTs and ITTs were conducted at the end of this experiment as in previous reports [22,23]. For GTT, mice were fasted overnight for 12 h, and glucose (2 g/kg body weight) was injected intraperitoneally. For ITT, insulin (0.75 U/kg body weight) was injected intraperitoneally without fasting. Mice were sacrificed after RA treatment. The blood samples were collected for the determination of glucose values using an Accu-check glucometer (Roche Diagnostics, Basel, Switzerland). Insulin levels in the serum were measured using an ELISA kit (Mouse Insulin ELISA, Mercodia, Sweden), according to the standard procedure [24]. The intra- and inter-assay coefficient of variation for the insulin ELISA kit were <4% and <6%, respectively. After 14 weeks of HFD feeding, the mice were sacrificed, and the serum was collected. Serum total cholesterol (TC), alanine aminotransferase (ALT), and aspartate aminotransferase (AST) levels were measured using Roche Modular P800 Automatic Analyser (Roche, Basel, Switzerland).

### Histopathological examination

Haematoxylin and eosin (H&E) staining of adipose and liver tissues was performed as previously reported [25,26]. Intrahepatic triglyceride (TG) content was measured by colorimetric methods (Triglyceride Quantification Kit, BioVision, USA) [27].

## Statistical analysis

Each experiment was repeated at least three times. All data are presented as mean ± SEM. One-way ANOVA following the post-hoc tests by Tukey was used to compare multiple groups, and differences (P < 0.05) have been labelled with different letters; same letters mean no significance(P > 0.05). The unpaired two-tailed Student’s t-test was used for comparisons between two groups and significances were marked with * (P < 0.05), ** (P < 0.01), *** (P < 0.001), and **** (P < 0.0001).

## Data Availability

The datasets used and/or analyzed during the current study are available from the corresponding author on reasonable request.

## List of abbreviations

retinoic acid: RA

non-alcoholic fatty liver disease: NAFLD

nonalcoholic steatohepatitis: NASH

white adipose tissue: WAT

brown adipose tissue: BAT

high-fat diet: HFD

respiratory exchange ratio: RER

oxygen consumption rate: VO_2_

carbon dioxide production rate: VCO_2_

total cholesterol: TC

alanine aminotransferase: ALT

aspartate aminotransferase: AST

## References

[1] Zhu S, Wu Y, Ye X, et al. FGF21 ameliorates nonalcoholic fatty liver disease by inducing autophagy. Mol Cell Biochem. 2016;420(1–2):107–119. 27435856 10.1007/s11010-016-2774-2

[2] Zhu SL, Zhang Z-Y, Ren G-P, et al. [Therapeutic effect of fibroblast growth factor 21 on NAFLD in MSG-iR mice and its mechanism]. Yao Xue Xue Bao. 2013;48(12):1778–1784. 24689234

[3] Amengual J, García-Carrizo F, Arreguín A, et al. Retinoic Acid Increases Fatty Acid Oxidation and Irisin Expression in Skeletal Muscle Cells and Impacts Irisin In Vivo. Cell Physiol Biochem. 2018;46(1):187–202. 29587291 10.1159/000488422

[4] Berry DC, Noy N. All-trans-retinoic acid represses obesity and insulin resistance by activating both peroxisome proliferation-activated receptor beta/delta and retinoic acid receptor. Mol Cell Biol. 2009;29(12):3286–3296. 19364826 10.1128/MCB.01742-08 PMC2698724

[5] Geng C, et al. Retinoic acid ameliorates high-fat diet-induced liver steatosis through sirt1. Science China-Life Sciences. 2017;60(11):1234–1241. 28667519 10.1007/s11427-016-9027-6

[6] Mercader J, Ribot J, Murano I, et al. Remodeling of white adipose tissue after retinoic acid administration in mice. Endocrinology. 2006;147(11):5325–5332. 16840543 10.1210/en.2006-0760

[7] Tourniaire F, Musinovic H, Gouranton E, et al. All-trans retinoic acid induces oxidative phosphorylation and mitochondria biogenesis in adipocytes. J Lipid Res. 2015;56(6):1100–1109. 25914170 10.1194/jlr.M053652 PMC4442868

[8] Saeed A, Dullaart R, Schreuder T, et al. Disturbed vitamin a metabolism in non-alcoholic fatty liver disease (NAFLD). Nutrients. 2017;10(1):29. 10.3390/nu10010029 PMC5793257 29286303

[9] Russo L, Lumeng CN. Properties and functions of adipose tissue macrophages in obesity. Immunology. 2018;155(4):407–417. 30229891 10.1111/imm.13002 PMC6230999

[10] Wronska A, Kmiec Z. Structural and biochemical characteristics of various white adipose tissue depots. Acta Physiol (Oxf). 2012;205(2):194–208. 22226221 10.1111/j.1748-1716.2012.02409.x

[11] van der Poorten D, Milner K-L, Hui J, et al. Visceral fat: a key mediator of steatohepatitis in metabolic liver disease. Hepatology. 2008;48(2):449–457. 18627003 10.1002/hep.22350

[12] Wang B, Fu X, Liang X, et al. Retinoic acid induces white adipose tissue browning by increasing adipose vascularity and inducing beige adipogenesis of PDGFRα(+) adipose progenitors. Cell Discov. 2017;3(1):17036. 29021914 10.1038/celldisc.2017.36 PMC5633810

[13] Suárez-Zamorano N, Fabbiano S, Chevalier C, et al. Microbiota depletion promotes browning of white adipose tissue and reduces obesity. Nat Med. 2015;21(12):1497–1501. 26569380 10.1038/nm.3994 PMC4675088

[14] Chusyd DE, et al. Relationships between rodent white adipose fat pads and human white adipose fat depots. Front Nutr. 2016;3:10. 27148535 10.3389/fnut.2016.00010 PMC4835715

[15] Benbrook DM, et al. History of retinoic acid receptors. Subcell Biochem. 2014;70:1–20. 24962878 10.1007/978-94-017-9050-5_1

[16] Yamauchi T, Waki H, Kamon J, et al. Inhibition of RXR and PPARgamma ameliorates diet-induced obesity and type 2 diabetes. J Clin Invest. 2001;108(7):1001–1013. 11581301 10.1172/JCI12864 PMC200951

[17] Di Masi A, Leboffe L, De Marinis E, et al. Retinoic acid receptors: from molecular mechanisms to cancer therapy. Mol Aspects Med. 2015;41:1–115. 25543955 10.1016/j.mam.2014.12.003

[18] Villarroya F. Differential effects of retinoic acid on white and brown adipose tissues. an unexpected role for vitamin A derivatives on energy balance. Ann N Y Acad Sci. 1998;839:190–195. 9629149 10.1111/j.1749-6632.1998.tb10757.x

[19] Zelent A, Krust A, Petkovich M, et al. Cloning of murine alpha and beta retinoic acid receptors and a novel receptor gamma predominantly expressed in skin. Nature. 1989;339(6227):714–717. 2544807 10.1038/339714a0

[20] Lee SA, Jiang H, Feranil JB, et al. Adipocyte-specific expression of a retinoic acid receptor α dominant negative form causes glucose intolerance and hepatic steatosis in mice. Biochem Biophys Res Commun. 2019;514(4):1231–1237. 31109648 10.1016/j.bbrc.2019.05.075

[21] Wolins NE, Quaynor BK, Skinner JR, et al. OP9 mouse stromal cells rapidly differentiate into adipocytes: characterization of a useful new model of adipogenesis. J Lipid Res. 2006;47(2):450–460. 16319419 10.1194/jlr.D500037-JLR200

[22] Bargut TCL, Mandarim-de-lacerda CA, Aguila MB. A high-fish-oil diet prevents adiposity and modulates white adipose tissue inflammation pathways in mice. J Nutr Biochem. 2015;26(9):960–969. 25997866 10.1016/j.jnutbio.2015.04.002

[23] Chakraborty A, Koldobskiy MA, Bello NT, et al. Inositol pyrophosphates inhibit akt signaling, thereby regulating insulin sensitivity and weight gain. Cell. 2010;143(6):897–910. 21145457 10.1016/j.cell.2010.11.032 PMC3052691

[24] Shang H, Sun J, Chen YQ. Clostridium butyricum CGMCC0313.1 modulates lipid profile, insulin resistance and colon homeostasis in obese mice. PLoS One. 2016;11(4):e0154373. 27123997 10.1371/journal.pone.0154373 PMC4849746

[25] Kim SY, Wi H-R, Choi S, et al. Inhibitory effect of anthocyanin-rich black soybean testa (Glycine max (L.) Merr.) on the inflammation-induced adipogenesis in a DIO mouse model. J Funct Foods. 2015;14:623–633.

[26] He Z, Zhu HH, Bauler TJ, et al. Nonreceptor tyrosine phosphatase Shp2 promotes adipogenesis through inhibition of p38 MAP kinase. Proc Natl Acad Sci U S A. 2013;110(1):E79–88. 23236157 10.1073/pnas.1213000110 PMC3538237

[27] Rodriguez A, Moreno NR, Balaguer I, et al. Leptin administration restores the altered adipose and hepatic expression of aquaglyceroporins improving the non-alcoholic fatty liver of ob/ob mice. Sci Rep. 2015;5(1):12067. 26159457 10.1038/srep12067 PMC4498231

